# Susceptibility to cigarette smoking and associated factors among high school students in western Ethiopia

**DOI:** 10.1186/s13104-018-3734-6

**Published:** 2018-08-30

**Authors:** Firew Tekle Bobo, Palanichamy Thanasekaran, Agin Jiju Rajamony Joice, Birhanu Yadecha, Animut Alebel

**Affiliations:** 1 Department of Public Health, Wollega University, Nekemte, Oromia Ethiopia; 2 Department of Nursing, Wollega University, Nekemte, Oromia Ethiopia; 3 Department of Nursing, Ambo University, Waliso, Oromia Ethiopia; 4 Department of Nursing, Debre Markos University, Debre Markos, Ethiopia

**Keywords:** Cigarette smoking, Susceptibility to cigarette smoking, Adolescents, Western Ethiopia

## Abstract

**Objective:**

Tobacco smoking is one of the leading causes of preventable premature death worldwide. Adolescence is a common period at which most of the established smokers start experimenting and smoking. The aim of the study was to determine the prevalence of susceptibility to cigarette smoking and associated factors among high school students in western Ethiopia.

**Result:**

The prevalence of susceptibility to cigarette smoking among the study participants was 16.9%. Two-third (65.9%, 95% CI; 62.77, 68.87) of the students reported that they are exposed to second hand smoking in public areas. Students, whose father smoked (OR 2.76, 95% CI [1.26, 6.09]), whose friends smoked (OR 3.73 95% CI [1.57, 8.90]). Adolescents who have the perception that boys who smoke are attractive (OR 2.26, 95% CI [1.24, 4.09]) and smoking cigarettes makes young people look cool (OR 1.47, 95% CI; [1.01, 2.17]) were more likely to be susceptible to smoking. Having the knowledge that tobacco smoking is harmful (OR .43, CI 95% [.28, .67]) to health was found to be a protective factor against susceptibility to smoking cigarette.

## Introduction

The major health events; disease, disability and fatality in the world can be attributed to a selected number of the most lethal risks to human health and tobacco use takes the driving seat among the top ten leading health risk factors in worldwide [1]. Smoking is one of the few risks, which causes a large number of early death and is responsible for a very large share of the global burden of disease [2–6]. Cigarette smoking has dramatically reduced in recent decades in developed countries. However, it is extremely increasing in less developed countries [7].

Smoking not only harms the smokers, but is also a certain health risk for the passive smokers and exposure to tobacco products during pregnancy can adversely affect development of the fetus. Each year up to 7 million people die from tobacco use, more than 6 million die from direct smoking, while an estimated 820,000 people die from secondary smoking [8].

Even though the dangers of smoking affect all socioeconomic levels, low-income households and countries are the once experiencing the most harm. Majority of world’s population lives in middle and low-income countries where the overall smoking rate is raising, but which have limited resources to respond to the health, economic and social problems caused by tobacco use [5, 9, 10].

In most cases, established tobacco smokers are exposed to tobacco products at their early adolescence. Tobacco control initiatives would yield more gains by giving priority to prevention of adolescents from smoking [11, 12].

There is general agreement that there are at least two major behavioral transitions involved in becoming a smoker: the transition from never smoker to experimenter and from experimenter to established smoker [13]. Thus, identification of smoking susceptible individuals and its determinants is important in the efforts to reduce future smoking prevalence.

Prevalence of smoking is low in Ethiopia. According to 2016 Ethiopian demographic health survey, prevalence of smoking is 1% among women and 4% among men [14]. Ethiopia has recently signed and deployed the tobacco control strategy forwarded by WHO [15]. It is very important that the prevalence of smoking remains low. Therefore, the aim of this study is to estimate prevalence of susceptibility to smoke among Nekemte, Ethiopia and identify factors associated with it.

## Main text

### Methods

#### Study setting/subjects

A school based cross sectional study design was employed. There were four public and three private high schools in Nekemte city. The sample was pulled in two stages. In the first stage, two public and two private high schools were randomly selected. In the second stage, 678 students were selected from two public high schools. While, 410 students were from two private high schools. Data were collected from all the students available the day of data collection. The data were collected in classrooms during break time.

#### Measurement

Self-administered questionnaires were used to collect the data. The study instrument was majorly derived from Global Youth Tobacco Survey (GYTS) questionnaire [16]. The survey questionnaire was first developed in English, translated into Afaan Oromo, and then back-translated. Minor modifications were made to ensure the questionnaire captures the local context.

Susceptibility to smoking among never smokers was measured using two sets of questions [13]. The first question was “Do you think you will be smoking cigarettes in the next 1 year from now?” followed by response options of definitely not, probably not, probably yes and definitely yes. The second question was, “If one of your best friends were to offer you a cigarette, would you smoke it?” followed by the same set of options as the first question. Lack of commitment to the response of “definitely not” for either of the questions was considered as having the intention to smoke cigarette.

The independent variables were organized and collected in three sections. The first section was sociodemographic characteristics, which included age, sex, grade, residence, pocket money, and educational status of the parents. The second section was concerned with perceptions towards tobacco smoking. The third section measures susceptibility and practice of smoking among adolescents.

#### Data processing and analysis

Data were first cleaned and entered into Epi-info (V-3.5.2) and then exported to STATA 13 for further analysis. Descriptive statistics were computed and presented.

The outcome of the study was susceptibility to cigarette smoking. To identify predictors of susceptibility to cigarette smoking we first conducted bivariate regressions with each potential covariate. Then we fitted multivariate logistic model, with 95% confidence interval. Results were then presented as adjusted odds ratios (OR).

#### Ethical considerations

Ethical clearance was obtained from Wollega University ethical review board and then written permission letter from the school administrations were granted. Administrations of the selected schools were informed of the study objectives and protocols.

Informed consent was obtained from all the participants aged 18 or above or from parents/legal guardians of the under aged respondents. The respondents were informed of their right to abstain from participating or to withdraw at any time without any condition. Furthermore, measures ensuring information confidentiality and contact details for the study coordinator for any questions or concerns were provided.

### Results

#### Descriptive for sociodemographic

Majority of the respondents (57.9%) were male, 91.3% of them from urban area. Majority (58.7%) of the respondents were grade 9, followed by 26.7% grade 10 students. About thirty-nine percent of respondent’s father were University level educated, while 26% respondent’s mothers were primary or less than primary level educated. The majority of the respondents (92.6%) reported that none of their family members is a smoker (see Table 1).

**Table 1:** Sociodemographic characteristics of the respondents (N = 958)

Characteristics	Frequency	Percent
Sex		
Male	555	57.9
Female	403	42.1
Age		
14	17	1.8
15	192	20.0
16	324	33.8
17	196	20.5
18	166	17.3
19	63	6.6
Educational status		
Grade 9	562	58.7
Grade 10	115	12.0
Grade 11	256	26.7
Grade 12	25	2.6
Education status of father		
Elementary school or less	164	17.1
Middle school	203	21.2
High school	218	22.8
University	373	38.9
Education status of mother		
Elementary school or less	259	27.0
Middle school	246	25.7
High school	202	21.1
University	251	26.2
Family/parental smoking		
None	887	92.6
Sibling	29	3.0
Father	35	3.7
Mother	7	.7
Friends smoking		
None	772	80.6
Some	154	16.1
Most or all smoking	32	3.3
Total	958	100.0

#### Susceptibility to and practice of cigarette smoking

Table 2 reports on smoking status of the study participants. The findings of this study show that 162 (16.9%, 95% CI, 15–19) never smokers, are susceptible to cigarette smoking. More than one-third (37.5%, 95% CI; 34.4, 40.63) of the students reported that they are exposed to second-hand smoking at home. Two-third (65.9%, 95% CI; 62.77, 68.87) of the students also reported that they are exposed to second hand smoking in public areas.

**Table 2:** Perception and practice of cigarette smoking (N = 958)

Characteristics	Frequency	Percent	Confidence interval	
			Lower	Upper
Would you be smoking cigarette 1 year from now				
Definitely not	786	82.0	79.47	84.43
Probably not	36	3.8	2.65	5.16
Probably yes	104	10.9	8.96	13
Definitely yes	32	3.3	2.29	4.68
Accepting cigarette offered by one of best one’s friends smoking				
Definitely not	790	82.5	79.9	84.82
Probably not	67	7.0	5.46	8.79
Probably yes	59	6.2	4.72	7.87
Definitely yes	42	4.4	3.17	5.88
Tried cigarette smoking, even one or two puffs				
No	797	83.2	80.67	85.51
Yes	161	16.8	14.49	19.33
Second hand smoking at home				
No	599	62.5	59.37	65.6
Yes	359	37.5	34.4	40.63
Second hand smoking in public places				
No	327	34.1	31.13	37.23
Yes	631	65.9	62.77	68.87

#### Predictors of susceptibility to smoke cigarette

Table 3 shows factors associated with susceptibility to cigarette smoking among the students. Having pocket money lead to a higher risk of susceptibility to smoking (OR 1.38, 95% CI 1.18, 1.62). Among family members, father’s smoking was associated with increased susceptibility to smoking (OR 2.76, 95% CI 1.26, 6.09). The youth were also more likely to be susceptible to smoking if some (OR 2.35, 95% CI 1.50, 3.69) or most (OR 3.73 95% CI 1.57, 8.90) of their friends are smoking cigarette compared to none of their friend’s smoking. The youth who have the perception that boys who smoke are attractive (OR 2.26, 95% CI 1.24, 4.09) and smoking cigarettes makes young people look cool (OR 1.47, 95% CI; 1.01, 2.17) were more likely to be susceptible to smoking.

**Table 3:** Factors associated with susceptibility to smoking among the high school students from Nekemte western Ethiopia

	Crude		Adjusted	
	P-value	OR	P-value	OR
Age	.009	1.18 (1.04, 1.35)	.096	1.14 (.98, 1.33)
Pocket money	.000	1.43 (1.25, 1.64)	.000	1.38 (1.18, 1.62)
Family smoked				
None	1.00		1.00	
Sibling	.007	2.98 (1.36, 6.56)	.317	1.59 (.64, 3.98)
Father	.000	4.25 (2.12, 8.51)	.012	2.76 (1.26, 6.09)
Mother	.009	7.56 (1.67, 34.16)	.825	1.24 (.18, 8.62)
Friends smoked				
None	Ref.		Ref.	
Some	.000	3.29 (2.20, 4.92)	.000	2.35 (1.50, 3.69)
Most or all smoking	.000	10.51 (5.02, 21.98)	.003	3.73 (1.57, 8.90)
Think that boys who smoke have more friends				
Yes	.000	1.96 (1.39, 2.76)	.229	1.303 (.85, 2.01)
No	Ref.	1.00		
Think that like girls who smoke had more friends				
Yes	.001	1.85 (1.29, 2.63)	.260	1.29 (.83, 2.03)
No	Ref.		1.00	
Think that boys who smoke are attractive				
Yes	.000	4.37 (2.81, 6.79)	.007	2.26 (1.24, 4.09)
No	Ref.		1.00	
Think girls who smoke are attractive				
Yes	.000	3.86 (2.36, 6.31)	.773	1.11 (.56, 2.18)
No	Ref.		1.00	
Think that tobacco smoking is harmful to health				
Yes	.011	.55 (.35, .87)	.482	.825 (.48, 1.41)
No	Ref.		1.00	
Think people can get addicted to tobacco smoking				
Yes	.000	.43 (.28, .67)	.003	.47 (.29, .78)
No	Ref.		1.00	
Smoking cigarettes makes young people look cool				
Yes	.000	2.03 (1.44, 2.86)	.047	1.47 (1.01, 2.17)
No	Ref.		1.00	

### Discussion

This study aimed to assess susceptibility cigarette smoking and associated factors among high school students in Nekemte, western Ethiopia. One-sixth (16.9%, 95% CI 15–19) of the study participants were susceptible to cigarette smoking. This finding is less than the study from Poland (22%) and in line with the study from Malaysia (16%) [17, 18]. On the other hand, this finding was found to be higher than the study from Thailand (9%), Taiwan (11%) and Pakistan (12%) [19, 20]. The differences between the studies concerning the proportion of those susceptible to smoking can be due to: social and cultural norms, and the fact that smoking is increasing in low and middle income countries [21].

This study has identified various factors that influence susceptibility to smoking among young people from low-income countries such as Ethiopia. Our result indicates that there is a strong association between parents’ and friends’ smoking status and smoking initiation among adolescents. Friends’ smoking is a strong predictor of susceptibility to smoking among the never smokers. This can be explained by the assumption that people tendence to choose their friends based on shared characteristics, including tobacco smoking. Fathers’ smoking status was also an important predictor of susceptibility to smoking [11, 17, 22].

Misperceptions among youth such as, boys who smoke are more attractive than those who do not and smoking makes young people look cool were significantly related to smoking susceptibility. This implies that peer context in which the youth find themselves plays a crucial role in smoking initiation and justifies further consideration to antismoking activities devoted to young people [12, 23, 24]. This study is the first one to report association of various risk factors with susceptibility to smoking from Ethiopia. Further study will be needed to confirm these findings and determine whether these findings can be generalized to our national population.

The current prevalence of tobacco smoking is low in Ethiopia. However, the findings of this study show that there is a very high probability that the prevalence of smoking may increase in the near future. Interventions that focus and can raise awareness of adolescents on the harms of smoking should be provided.

### Conclusion

Youth nonsmokers are the target population for smoking prevention activities. Our study has revealed important factors that are common among smoking-susceptible participants. Having pocket money, a father who smoked, report that their friends smoked, perception that boys who smoke are more attractive, and the believe that smoking makes young people look cool believe. On the other hand, the perception that tobacco smoking is harmful to health was only the protective factor against susceptibility to smoking cigarette.

### Limitations of the study

Firstly, generalization of our study should be taken cautiously. As our survey is not representative of all adolescents or age groups, because it was conducted among students and adolescents enrolled in secondary- or high schools.

Moreover, all estimates in our study were based on self-reports, which might be affected by reporting bias. The practice of smoking or having the intention to smoke may not be acceptable socially. As a result, the reports might also be affected by social desirability bias. The other limitation of our study was that our analysis did not control for other substances use such as alcohol or illicit drugs, which are also indicated to be associated with smoking. Despite the mentioned limitations, the current study provides a valuable insight into the prevalence and factors associated with susceptibility to smoking among youth in western Ethiopia.
